# A Phase 1 Trial of pharmacologic interactions between transdermal selegiline and a 4-hour cocaine infusion

**DOI:** 10.1186/1472-6904-9-13

**Published:** 2009-08-01

**Authors:** Debra S Harris, Thomas Everhart, Peyton Jacob, Emil Lin, John E Mendelson, Reese T Jones

**Affiliations:** 1 Department of Psychiatry, University of Cincinnati, OH, USA; 2 Cincinnati VA Medical Center, OH 45220, USA; 3 Drug Dependence Research Center, University of California, San Francisco, USA; 4 Drug Studies Unit, University of California, San Francisco, USA; 5 Addiction Pharmacology Research Laboratory, California Pacific Medical Center Research Institute, San Francisco, CA, USA

## Abstract

**Background:**

The selective MAO-B inhibitor selegiline has been evaluated in clinical trials as a potential medication for the treatment of cocaine dependence. This study evaluated the safety of and pharmacologic interactions between 7 days of transdermal selegiline dosed with patches (Selegiline Transdermal System, STS) that deliver 6 mg/24 hours and 2.5 mg/kg of cocaine administered over 4 hours.

**Methods:**

Twelve nondependent cocaine-experienced subjects received deuterium-labeled cocaine-d5 intravenously (IV) 0.5 mg/kg over 10 minutes followed by 2 mg/kg over 4 hours before and after one week of transdermal selegiline 6 mg/24 hours. Plasma and urine were collected for analysis of selegiline, cocaine, catecholamine and metabolite concentrations. Pharmacodynamic measures were obtained.

**Results:**

Selegiline did not change cocaine pharmacokinetic parameters. Selegiline administration increased phenylethylamine (PEA) urinary excretion and decreased urinary MHPG-sulfate concentration after cocaine when compared to cocaine alone. No serious adverse effects occurred with the combination of selegiline and cocaine, and cocaine-induced physiological effects were unchanged after selegiline. Only 1 peak subjective cocaine effects rating changed, and only a few subjective ratings decreased across time after selegiline.

**Conclusion:**

No pharmacological interaction occurred between selegiline and a substantial dose of intravenous cocaine, suggesting the combination will be safe in pharmacotherapy trials. Selegiline produced few changes in subjective response to the cocaine challenge perhaps because of some psychoactive neurotransmitters changing in opposite directions.

## Background

The acute administration of cocaine produces euphoria and chronic administration produces hedonic dysregulation, which are both believed to be mediated by dopaminergic systems [1]. Chronic cocaine use-induced dysregulation of the brain dopamine system may mediate cocaine reward, craving and relapse [2]. One approach to treating this dysregulation has been the use of medications that increase dopamine activity. Selegiline, a relatively selective monoamine oxidase (MAO)-B inhibitor at lower doses has been investigated as a pharmacological treatment for cocaine dependence.

Selegiline, approved for the treatment of Parkinson's Disease, inactivates MAO-B irreversibly and increases its substrate dopamine in the synapse; reactivation requires at least one to two weeks [3]. A placebo-controlled, double-blind treatment trial using selegiline 10 mg per day orally for eight weeks (a dose in the range selective for MAO-B) suggested selegiline may be useful for the treatment of cocaine dependence, based on decreases in quantitative urine concentrations of the metabolite (benzoylecgonine) and improved self- and investigator-rated global clinical status (prepublication data, NIDA-MDD). However, a later study using the selegiline transdermal system (STS) was not as encouraging [4].

Transdermal selegiline is well tolerated, avoids the erratic bioavailability from extrahepatic metabolism in the gut [5] and extensive first pass metabolism [6], may decrease the risk of hypertensive crisis ("cheese effect"), and may decrease some of the side effects by decreasing generation of selegiline's major metabolites, l-methamphetamine and l-amphetamine [7].

Several studies have evaluated the safety of selegiline and cocaine co-administration [8-11]. These studies all used a single dose of 20 or 40 mg of intravenous (IV) cocaine for each cocaine challenge session following daily dosing of selegiline orally [8-10] or transdermally [11]. Our study also evaluated the pharmacologic interactions between 7 days of patches delivering 6 mg/24 hours of selegiline transdermally and cocaine. However, because persons with cocaine dependence often use repeated larger doses of cocaine during a period of use, we administered a larger dose of cocaine (2.5 mg/kg) as an infusion over a longer period of time (4 hours) to more closely simulate the doses and duration of use reported by many patients. This increased exposure to cocaine allowed assessment of drug-drug interactions at drug levels likely to be encountered in clinical practice.

Because of the importance of dopamine to the rewarding effects of drugs, concentrations of dopamine or its metabolites may help explain changes in the subjective effects of cocaine given during selegiline administration. Homovanillic acid (HVA) is a major metabolite of dopamine released in the brain, and although plasma concentrations of this metabolite are influenced by other factors, it may be a useful indicator of brain dopamine activity [12].

In addition to inhibiting the reuptake of dopamine, cocaine is also a potent inhibitor of norepinephrine reuptake. Measurement of urinary 3-methoxy-4-hydroxyphenylglycol (MHPG), the main excretory product of norepinephrine, has been used as an index of the release of brain norepinephrine. Because MHPG is conjugated to MHPG-S in the central nervous system prior to excretion [13] we chose to analyze for only the conjugated MHPG-S in order to more specifically focus on brain norepinephrine activity.

Cocaine would be expected to increase brain norepinephrine turnover with a subsequent increase in urine levels of MHPG-S. Acute doses of selegiline should also increase synaptic norepinephrine and corresponding urinary MHPH-S levels, unless significant MAO-A inhibition is present, preventing the metabolism of norepinephrine to MHPG. However, with sustained selegiline dosing, norepinephrine depletion could occur and MHPG-S levels could decline to baseline (or even below). Assessing the effects of cocaine before and after treatment with selegiline could provide information about changes in cocaine-induced norepinephrine regulation.

It is not clear how much, if any, MAO-A inhibition occurs after treatment with 6 mg/24 hours of STS or what risk this poses, particularly when other drugs or foods that increase catecholamine activity are co-administered. Hypertensive crises have been reported with MAO-A inhibitors and sympathomimetic amines, such as phenylpropanolamine [14]. The interactions between MAO-A inhibitors and cocaine administered are largely unexplored. However, cocaine during use of a MAO-A inhibitor did not produce a hypertensive crisis in a preclinical study [15] and a case report [16]. Since hypertensive crises do not occur in every case, surrogate measures would be important. Therefore, measurement of dopaminergic and noradrenergic tone may be important in predicting toxic events. We measured dopaminergic tone with the surrogate marker of plasma HVA, the major brain metabolite of dopamine, and serum levels of prolactin, a hormone whose secretion is inhibited by dopamine. The surrogate for noradrenergic tone was urine concentrations of MHPG-S. In order to assess the degree of MAO-A and MAO-B inhibition, we assayed concentrations of a substrate of MAO-B, phenylethylamine (PEA) [17-19]. PEA would be expected to increase with MAO-B inhibition, and concentrations of MHPG-S, which is a metabolite of norepinephrine produced by MAO-A primarily in the brain [20], would be expected to decrease. Since MHPG-S is both an indicator of brain norepinephrine activity and a metabolite of MAO-A, the pattern of change (after initiation of selegiline administration and after cocaine administration) can help define mechanistic interactions.

This paper reports pharmacological interactions between cocaine and selegiline. We describe effects on dopamine, norepinephrine, MAO-A, and MAO-B activity, and on physiological and subjective effects of cocaine before and after one week of transdermal selegiline.

## Methods

### Subjects

Subjects were recruited through newspaper advertisements asking for volunteers to participate in studies of cocaine effects. Subjects gave written informed consent and were paid for participating. The protocol was approved by the local Institutional Review Board and carried out in compliance with the Declaration of Helsinki.

Inclusion criteria were as follows: age 21–45, ability to give informed consent, previous experience with smoked or intravenous cocaine and continuing cocaine use (ranging from once a week to once in the last 6 months), good health, and within 15% of ideal body weight. Subjects were excluded for current dependence on any drug (except caffeine or nicotine) as defined by the *Diagnostic and Statistical Manual of Mental Disorders, Fourth Edition *criteria, pregnancy, or sensitivity to selegiline or related medications. Those interested in treatment or counseling were given appropriate referral information at the conclusion of the study.

Potential subjects were screened to eliminate those with significant medical or psychiatric illnesses. Evaluation included a comprehensive history and physical examination; an electrocardiogram; and laboratory tests, including hepatitis C serology, EKG, pregnancy screen, and HIV test.

### Study Medications

Deuterium labeled cocaine (cocaine-d5) for intravenous infusions was synthesized and purified [21] from USP grade cocaine hydrochloride (as outlined in IND #51,280). The UCSF Investigational Pharmacy prepared stock solutions of millipore filtered cocaine-d5 HCL in preservative-free normal saline (NS) from crystalline cocaine-d5 HCL. Cocaine-d5 was diluted for human administration in sterile 0.9% sodium chloride. A sample from each dose actually administered was assayed by gas chromatography/mass spectrometry (GC/MS) to evaluate concentration, stability, and quality control.

The STS (IND #42,302, 50,279, 46,944) 6 mg/24 hours patch was applied to the upper torso daily for 10 days, just below the fossa axillaris on the nondominant side, and was kept on for 24 hours until application of the next patch [22]. During both the inpatient and outpatient phases, selegiline patches were applied at 9 AM. Patches (lot #26E007D) were supplied by Somerset Pharmaceuticals, Inc., Tampa, Florida.

### Study Design

Subjects were admitted to the UCSF General Clinical Research Center (GCRC). The following day (day 1), the first cocaine infusion occurred; measures were collected for the next 3 days. Transdermal selegiline administration began on day 4 and the subject was discharged on day 5. Selegiline administration was continued on an outpatient basis. The subject was readmitted to the GCRC on day 10. The second cocaine infusion took place the following day (day 11) (2 hours after application of the selegiline patch) and measures were collected for the next 3 days. At this time the selegiline was discontinued and the subject was discharged.

In this open-label, unblinded study, a 0.5 mg/kg intravenous loading dose of cocaine-d5 HCl over 10 minutes was followed by a slow 4 hour constant rate cocaine-d5 infusion of 2.0 mg/kg(~175 mg for a 70 kg person). This produced a fairly stable plasma concentration over 4 hours. When this method of cocaine administration is utilized, a larger dose can be given over a longer period of time and approximates frequently reported patterns of cocaine use [23,24]. This method also allowed for close monitoring and termination of the infusion if untoward psychiatric or cardiovascular effects occurred.

The first infusion was performed 3 days prior to transdermal selegiline, and the second following one-week of transdermal selegiline administered as one STS patch per day. The order of cocaine infusions (i.e., following selegiline or prior to treatment) was fixed, as it may take several weeks for MAO-B activity to return to normal after use of the irreversible inhibitor selegiline [25].

### Dietary Restrictions

Subjects were instructed to eat a diet low in monoamines during the study. Breakfast and lunch were provided at the GCRC. For meals outside the GCRC, a list of high tyramine foods was provided with appropriate instructions. A daily food diary was collected and any lapses in low monoamine guidelines were discussed with the subjects.

### Blood and Urine Collection

For both cocaine infusions (sessions 1 and 2), plasma samples for cocaine and metabolites (~6 ml) were obtained 5 minutes before and 10 minutes, 0.5, 1, 1.5, 2, 4, 4.25, 4.5, 5, 6, 8, 12, 21, 27, 33, and 45 hours after each cocaine infusion. On day 4, a pre-dose plasma sample (~6 ml) was obtained 5 minutes before the selegiline patch was applied at 9 AM. On outpatient days (days 5 and 9), samples were taken 5 minutes prior to application of the selegiline patch to determine steady state trough concentrations (Css) of selegiline and metabolites.

On day 11, prior to the second cocaine infusion, samples (~6 ml) were obtained 5 minutes before application of transdermal selegiline. In times referenced to the cocaine infusion, samples for selegiline and metabolites (~6 ml) were obtained pre and at 2, 4, 6, 12, 18, 24, 36, and 48 hours after the second cocaine infusion. Additional samples for selegiline and metabolites were taken 5 minutes prior to the selegiline doses on the second and third day after the cocaine challenge (days 12 and 13).

Samples (~10 ml) for HVA and prolactin were collected at 5 minutes before cocaine infusion and 0.5, 1, 1.5, 2, 3 and 4 hours after the beginning of the infusion. To obtain accurate HVA measures, subjects remained recumbent for 30 minutes before and during the 4 hour infusion period. If subjects needed to void during this period, they used a hand-held urinal or a bedpan. Following collection, all samples were frozen at -70°C until analyzed.

When subjects were in the GCRC, all urine was collected and volumes recorded. Following each cocaine infusion, urine was collected in fractions from 0 to 24, 24 to 48, and 48 to 72 hours (timed from the start of the infusion). Samples were then frozen at -70°C until analyzed. Urine samples were analyzed for selegiline and metabolites, PEA, and MHPG-S. During the outpatient phase, a daily 9 AM (selegiline steady state) urine specimen was obtained and analyzed for selegiline and metabolites. A qualitative screen for drugs of abuse was obtained every other day for the entire study period.

### Pharmacodynamic Measures

#### Physiologic Measures

Heart rate, systolic and diastolic blood pressure were measured using a cardiovascular monitor (Dinamap by Critikon, Vital Signs Monitor 1846 SX). Rate pressure product was calculated as the product of systolic blood pressure and heart rate. Respiration rate was measured by counting the number of inhalations per minute. These vital signs were determined with the patient in a supine position for at least 10 minutes.

Heart rate, blood pressure, respiration rate, skin and core temperature were recorded at specific times during the study. During infusions, measures were obtained pre-dose and at 0.17, 0.5, 0.75, 1, 1.5, 2, 2.5, 3, 3.5, 4, 4.25, 4.5, 5, 6, 8, 12, 18, 24, 36, 48, and 72 hours after dosing. The electrocardiogram was monitored continuously during the infusion and for 4 hours after the termination of the intravenous cocaine infusion. EKG tracings were recorded at each data collection point.

Supervised administration of the transdermal selegiline began on day 4 and continued through day 12. Since orthostatic hypotension may occur with the administration of selegiline, orthostatic vital signs (i.e., blood pressure, heart rate, respiration rate, in supine and standing positions) were obtained prior to the transdermal selegiline and at 1 and 4 hours after application of the patch.

### Subjective Measures

Verbal ratings of global intoxication and craving (on a scale of 0 to 100) were obtained at the same time points as the physiologic measures, with 0 representing no drug effect and 100 representing the highest level of intoxication or craving.

Visual analog scales were used to rate the degree of drug liking by moving an arrow along a 10 cm line marked at opposite ends as 0 (not at all) and 100 (extreme) using a hand-held computer (Tandy 102). Items rated on the visual analog scale included "good drug effect" and "bad drug effect," and were obtained prior to cocaine infusion and at 0.17, 0.5, 0.75, 1, 1.5, 2, 2.5, 3, 3.5, 4, 4.25, 4.5, 5, 6, and 8 hours after dosing.

The Profile of Mood States (POMS) [26] assessed mood changes in Anger, Confusion, Fatigue, Tension, and Vigor on a scale from 0 to 4, where 0 indicated "no effect" and 4 indicates "extremely strong". Ratings were obtained prior to cocaine infusion and at 1, 3, and 6 hours after dosing.

### Determination of Metabolites in Plasma and Urine

Plasma concentrations of cocaine and benzoylecgonine were measured in our laboratory using a method we developed [27]. The method allows for simultaneous determination of cocaine, benzoylecgonine, and deuterium-labeled analogs [cocaine-d5 and benzoylecgonine-d5 (BE-d5)] by using combined gas chromatography-mass spectrometry (GC-MS) [27]. Differently labeled stable isotope analogs were used as internal standards. The calibration curves were from 5 to 2,000 ng/mL for cocaine-d5 and 10 to 2,000 ng/mL for BE-d5. Lower limits of quantitation (LLOQ) were 5 ng/ml (cocaine-d5) and 10 ng/ml (BE-d5) in plasma. Qualitative urine benzoylecgonine was measured by Northwest Toxicology Laboratory, Salt Lake City, Utah, using the Roche Diagnostics Online^® ^immunoassay.

Plasma and urine selegiline and metabolites were analyzed by liquid chromatography-tandem mass spectrometry (LC/MS/MS) (Dr. Emil Lim, UCSF Drug Studies Unit, San Francisco, CA). LLOQ was 0.025 ng/ml for selegiline and 0.100 ng/ml for its metabolites, n-desmethyselegiline, methamphetamine and amphetamine. Inter-day precision, defined by coefficient of variation (CV), ranged from 5.9 to 118% for selegiline, from 4.5 to 10.8% for n-desmethylselegiline, from 3.9 to 6.5% for methamphetamine, and from 3.9 to 16.1% for amphetamine, with an accuracy, defined by relative error (RE), of -5.4 to -6.5% for selegiline and metabolites.

The analytical method and assay validation for determination of concentrations of PEA in urine was by LC/MS/MS. LLOQ was 2.00 ng/ml for PEA. Interday CV ranged from 8.68 to 12.0%. RE ranged from -2.00 to +6.00%.

Serum prolactin was determined in duplicate by a direct (CT) RIA method using kits purchased from ICN Biomedical, Inc. (Costa Mesa, CA). The assay sensitivity was 0.1 ng/ml and the intra- and interassay CVs were 3.5 and 4.2%, respectively.

Plasma HVA was determined by HPLC. The HPLC method was adapted from that described by Minegishi and Ishizaki [28]. This method has a LLOQ of 2 ng/ml.

Concentrations of MHPG-S in urine were determined using LC-MS/MS [29]. After discharge from the GCRC on day 4 and during the outpatient period, subjects submitted a 9 AM urine sample every day immediately before application of the selegiline patch. As urinary creatinine excretion is directly related to glomerular filtration and renal blood flow, and urinary creatinine excretion is relatively constant (~1 gm/day), it can be used to estimate concentrations of a substance during a 24 hour urine collection period. Using ratios of the concentrations of MHPG-S to creatinine obtained from assays of these spot urine samples, MGPG-S concentrations during the outpatient period (when subjects would be unlikely to provide accurate 24 hour urine collections) were compared to those from urine samples on inpatient days.

### Pharmacokinetic Analysis

The plasma concentration-time profiles for cocaine and benzoylecgonine (BE) were analyzed by non-compartmental methods [30], utilizing the pharmacokinetic data analysis program WinNonlin Pro, version 3.0 [31]. Data from two subjects at two different time points were inconsistent with surrounding data, therefore, were considered outliers and not included in the pharmacokinetic data analysis.

The area under the plasma concentration-time curve was calculated using the linear trapezoidal rule up to the maximum plasma concentration, and thereafter using the logarithmic trapezoidal rule to the last measurable plasma concentration. The remaining area was extrapolated to infinity by dividing the last appropriate plasma concentration by the terminal exponential rate constant.

Maximum plasma concentration (C_max_) and time of maximum plasma concentration (T_max_) were determined by visual inspection of data. "Terminal" exponential half-life [(t1/2)z] was calculated from log-linear regression of "terminal" exponential phase data points. Cocaine clearance (CL) and terminal exponential volume of distribution (Vz) were calculated by conventional equations: CL = [Dose/Area under the curve (AUC)_0-∞_] and Vz = CL/λz, respectively, where λz ("terminal" exponential rate constant) = ln2/(t1/2)z. BE renal clearance was calculated as: amount in urine from 0 to 8 hours divided by AUC from 0–8 hours. Amount of BE in urine samples was calculated by multiplying concentration by volume.

### Statistical Analysis

For cocaine, descriptive statistics (mean, standard deviation (SD), percent coefficient of variation (% CV), median, minimum, and maximum) were calculated for each parameter over each session. Because in most cases one or both of the inherent assumptions of the ANOVA were violated (normalcy of distributions and equivalence of variances), the nonparametric Friedman test was utilized.

For selegiline, the statistical analyses were performed using the SAS Statistical Analysis System. All analyses employed a one-factor repeated-measures analysis of variance (ANOVA), with all parameters analyzed as their logarithmic (natural) transforms. If a day effect was found between days 9, 11, 12, and 13, a Tukey multiple comparison procedure was employed *posthoc *to compare the days in pairs. A test for linear trend was included. All probability estimates presented are based on the *Type III sums of squares*.

The time courses of physiologic and subjective data were analyzed by repeated measures analysis of variance (ANOVA). Treatment conditions (Sessions 1 and 2) and observation times (hours post-dose) were considered within-subject factors. Change scores (post-dose minus pre-dose values) were used in most analyses. Raw score were used where indicated. After a significant F test, pairwise comparisons of treatment conditions were performed using the least squares means analysis. Peak effects within a session were determined for each subject and each variable and analyzed using a one-factor repeated measures ANOVA. Effects were considered statistically significant at p = 0.05. Data were adjusted for sphericity using the Huynh-Feldt adjustment factor. Huynh-Feldt-corrected significance values are reported.

## Results

### Subjects

Eleven male and 1 female (mean age ± SD, 33 ± 7 years [range, 22–44 years]) completed the study. The ethnicity was predominately white with 9 Caucasians, one Asian, one Hispanic, and one African-American. Typical use was 2 lines to 1 gm of cocaine per occasion. Four typically smoked cocaine, five usually used the intranasal route, and three used both methods of administration (smoked and intranasal route). Two subjects left prior to completion of the study, one as a result of an erectile dysfunction that he believed had been caused by the study drug, and the other for personal reasons.

### Pharmacokinetic Results

**Figure 1:**
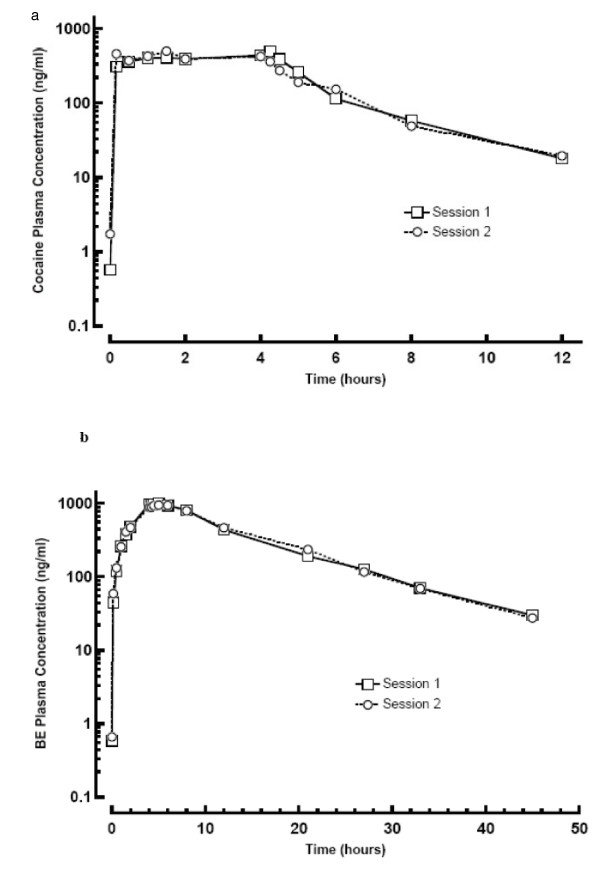
**Mean Plasma Cocaine (a) and Benzoylecgonine (b) Semi-logarithmic Plots for Cocaine Challenge Sessions 1 (before selegiline) and 2 (after selegiline)**.

The mean plasma concentration-time profiles of cocaine and BE are shown in Figure 1 (one outlier is removed). The BE AUC was approximately 5.9 times greater than that of cocaine. No effect of selegiline on cocaine or BE plasma pharmacokinetics was found following the removal of the two outliers. Table 1 summarizes calculated cocaine pharmacokinetic parameters and statistical results.

**Table 1:** Plasma Pharmacokinetic Parameters for Cocaine and Benzoylecgonine

**Substance** (mean ± SD)	**C_max_** **(ng/ml)**	**T_max_** **(hr)**	**AUC** **(0-t)** (ng*h/ml)	**λz** **(1/hr)**	**(t_1/2_)z** **(hr)**	**AUC** **(0-∞)** (ng*h/ml)	**AUC_ext_** **(%)**	**CL** **(ml/min/kg)**	**Vz** **(l/kg)**
**Cocaine** **Pre Selegiline** (session 1)	721 ± 773	2.66 ± 1.74	2640 ± 55.6	0.453 ± 27.9	1.71 ± 0.81	2680 ± 1470	1.40 ± 1.03	18.3 ± 6.17	2.49 ± 0.88

**Cocaine** **With Selegiline** (session 2)	640 ± 332	2.27 ± 2.04	2360 ± 499	0.456 ± 0.09	1.57 ± 0.30	2400 ± 487	1.78 ± 1.87	18.0 ± 3.34	2.44 ± 0.64

**Benzoyl-ecgonine** **Pre Selegiline** (session 1)	1045 ± 180	4.79 ± 0.50	13000 ± 3030	0.0928 ± 0.023	8.63 ± 5.36	13600 ± 3820	3.45 ± 6.45		

**Benzoyl-ecgonine** **With Selegiline** (session 2)	957 ± 184	5.04 ± 0.65	12900 ± 3760	0.0971 ± 0.024	7.52 ± 1.82	13200 ± 4180	2.21 ± 2.24		

* C_max _= maximum plasma concentration; T_max _= time of C_max_; AUC(0-t) = area under the plasma concentration-time curve from 0 time to the time of the last measurable plasma concentration; AUC(0-∞) = area under the plasma concentration-time curve from 0 to infinity; AUC_ext _(%) = percent of AUC(0-∞) determined by extrapolation; λz = "terminal" exponential rate constant; (t_1/2_)z = "terminal" exponential half-life; CL = clearance; Vz = "terminal" exponential volume of distribution; SD = standard deviation and %CV = percent coefficient of variation.

**Figure 2:**
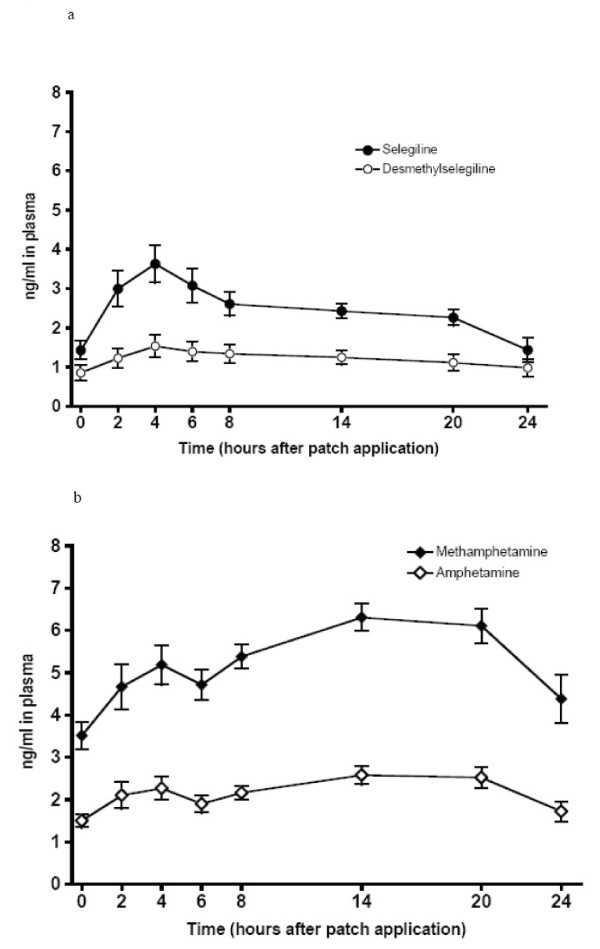
**Mean Plasma Concentrations of Selegiline and Metabolites during Session 2**. Cocaine infusion began 2 hours after the application of the selegiline patch. (a) Black Circle = Selegiline, Open Circle = Desmethylselegiline. (b) Black Diamond = Methamphetamine, Open Diamond = Amphetamine. Error bars = SE.

Mean plasma concentrations of selegiline and metabolites rose slightly after cocaine with the largest increase occurring 3 days after the cocaine infusion. However, the trough plasma concentrations of only l-methamphetamine and l-amphetamine increased over time from days 9, 11, 12, and 13 (p for linear trend <0.001 and <0.01, respectively). Figure 2 represents mean plasma concentrations of selegiline and metabolites immediately before and following the cocaine infusion.

Table 2 shows that the 24 hour urine collections (timed at the start of the cocaine infusion, 2 hours after application of transdermal selegiline) were not significantly different for selegiline or its metabolites for the 0–24, and 24–48 hours collection periods. The concentration of selegiline and each of its metabolites (desmethylselegiline, methamphetamine, and amphetamine) in the 48–72 hour collection period was less than that of the previous days. However, it should be noted that the selegiline daily patch was not applied the morning of the last day for the final two hours. Thus, less would be expected.

**Table 2:** Selegiline and Metabolites Excreted in the 24 Hour Urines from 0–24, 24–48, and 48–72 hours After Cocaine (Mean ± SD, N = 12)

**Drug Assayed**	**0–24 hours** **(day 11)**	**24–48 hours** **(day 12)**	**Statistical p Value** **0–24 vs. 24–48 hours**	**48–72 hours** **(day 13)**
Selegiline	0.109 ± 0.073	0.145 ± 0.138	0.34	0.063 ± 0.097
Desmethylselegiline	0.184 ± 0.128	0.188 ± 0.162	0.75	0.082 ± 0.086
l-Methamphetamine	6.26 ± 2.28	8.51 ± 5.69	0.20	4.83 ± 1.55
l-Amphetamine	2.34 ± 0.85	3.30 ± 2.34	0.18	2.15 ± 0.94

Infusion started on Day 11 at 11 AM, 2 hr after the 9 AM daily patch application.

No selegiline daily patch was applied at 9 AM on day 14.

### Pharmacodynamic Measures

#### Physiologic Measures

**Figure 3:**
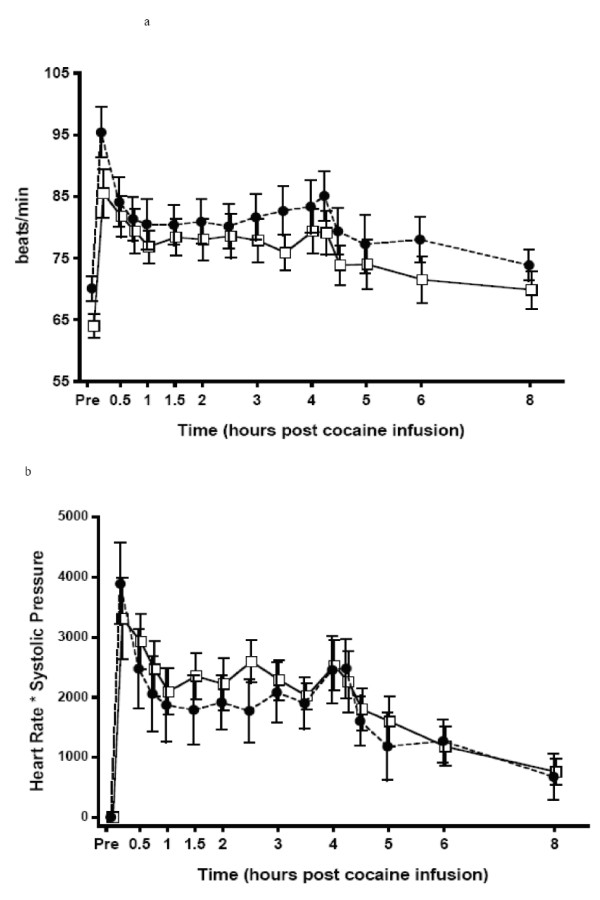
**Mean Heart Rate Readings (a) and Rate Pressure Product (b)**. Open Square = Session 1, Black Circle = Session 2. Error bars = SE.

Mean heart rate and rate pressure product after cocaine are shown in Figure 3. Compared to baseline values, systolic blood pressure, heart rate, and rate pressure product increased significantly (p < 0.01). In contrast, skin temperature decreased significantly (p < 0.01) during both challenge sessions. Neither peak effects (Table 3) nor physiological effects across time were affected by selegiline. Cocaine, either alone, or with selegiline did not alter diastolic blood pressure, respiration rate, and core temperature. Orthostatic blood pressure was within normal limits.

**Table 3:** Physiologic and Subjective Measures – Summary of Peak Changes (Mean ± SD for 12 Subjects)

	**Session 1**		**Session 2**	
**Measures**	**Peak** **Mean ± SD**	**Peak Time** **(Hr Postdose)**	**Peak** **Mean ± SD**	**Peak Time** **(Hr Postdose)**

**Physiologic Measures**				
Systolic Blood Pressure (mmHg)	17 ± 8	2.2 ± 1.9	17 ± 9	1.9 ± 1.7
Diastolic Blood Pressure (mmHg)	10 ± 5	2.8 ± 2.4	12 ± 6	2 ± 2
Heart Rate (beats/min)	26 ± 10	0.8 ± 0.9	27 ± 12	0.9 ± 1.7
Rate Pressure Product (SBP*HR)	4224 ± 1638	1.4 ± 1.5	4437 ± 2333	0.9 ± 1.6
Respiration Rate (inhal/min)	3.8 ± 4.0	0.9 ± 1.3	3.8 ± 3.6	0.4 ± 0.3
Skin Temperature (°C)	-5.4 ± 2.7	0.8 ± 0.4	-5 ± 3.2	1.0 ± 1.2
Core Temperature (°C)	0.7 ± 0.2	4.5 ± 1.6	0.7 ± 0.5	4.2 ± 1.9
				
**Subjective Measures**				
Global Intoxication (0–100)	57 ± 27	0.7 ± 1	53 ± 23	0.6 ± 0.9
Cocaine Craving (0–100)	33 ± 25	1.1 ± 1.3	20 ± 21	0.6 ± 0.7
Good Drug Effect (0–100)	66 ± 23	0.9 ± 1.5	60 ± 21	1 ± 1.3
Bad Drug Effect(0–100)	46 ± 31	1.9 ± 2.3	55 ± 25	1.4 ± 1.9
				
**POMS Scales**				
Tension (0–28)	7.5 ± 6.1	1 ± 0	4.8 ± 3.9	1 ± 0
Depression (0–64)	3 ± 6.5	3 ± 0	1.5 ± 2.4	3 ± 0
Anger (0–28)	2.8 ± 6.4	6 ± 0	-2.1 ± 5.7*	6 ± 0
Vigor (0–32)	-3.6 ± 3.4	6 ± 0	-3.9 ± 6.1	6 ± 0
Confusion (0–20)	2.5 ± 2.8	6 ± 0	2.7 ± 4.7	3 ± 0
Fatigue (0–28)	5.9 ± 5.9	6 ± 0	2.8 ± 5.3	6 ± 0

* Significantly different from Session 1 (p < 0.03).

Session 1: First cocaine infusion, performed on day 1, before selegiline administration.

Session 2: Second cocaine infusion, performed on day 11, following one week of selegiline administration by STS patch (6 mg/24 hours). Infusion began 2 hours after application of the selegiline daily patch.

### Subjective Measures

**Figure 4:**
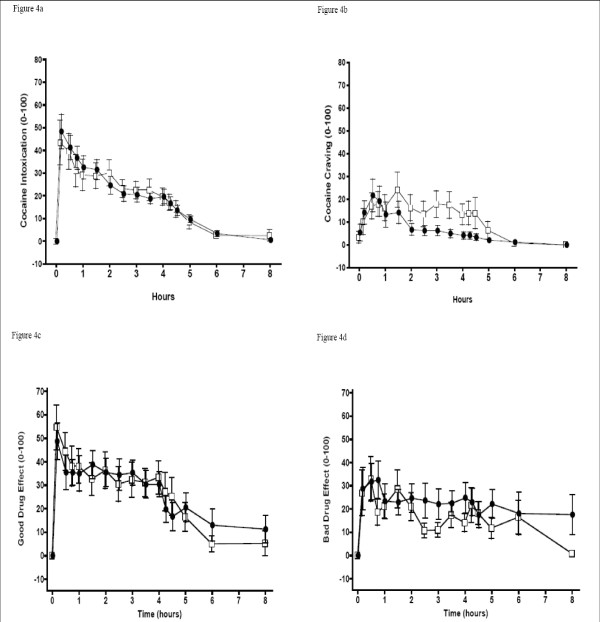
**Mean Subjective Measures: Verbal and Visual Analog Scale Ratings**. (a) Intoxication, (b) Craving, (c) Good Drug effect, (d) Bad Drug Effect. Open Square = Session 1, Black Circle = Session 2. Error bars = SE.

**Figure 5:**
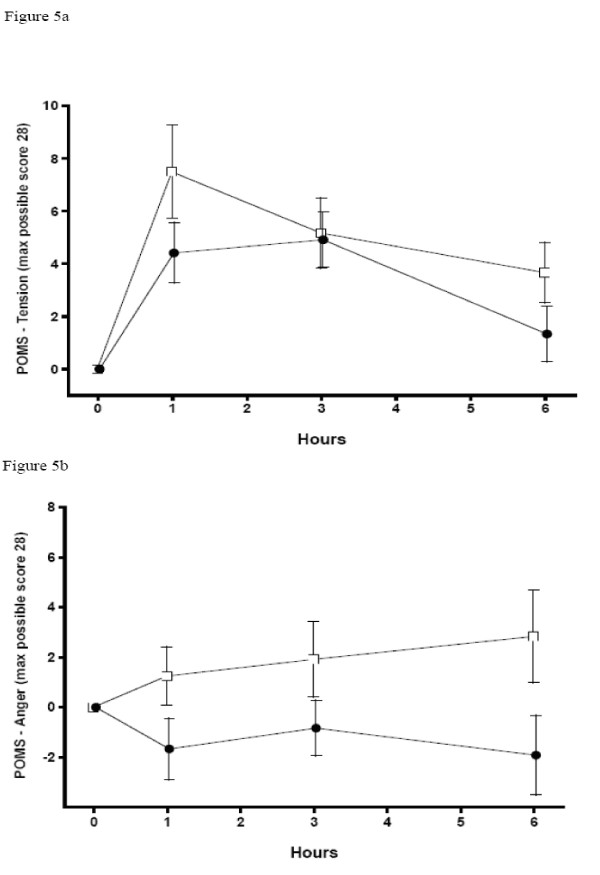
**Mean Subjective Measures: Profile of Mood States (POMS) Measures**. Open Square = Session 1, Black Circle = Session 2. Error bars = SE.

Mean verbal and visual analog subjective ratings across time are shown in Figure 4 and POMS scales "anger" and "tension" are in Figure 5. Compared to baseline, global intoxication, verbal ratings of craving, and "good drug effect" increased in both cocaine challenge sessions. Only the POMS "anger," "tension," and total score scales were significantly different (lower) during the second session (after selegiline). Verbal ratings of craving were also lower in the second session between 1.5 and 4.5 hours after cocaine (i.e., condition × time was significant).

Peak changes and peak times are shown in Table 3. No significant differences in peak ratings were found between sessions on the verbally reported or visual analog scales, although cocaine craving showed a trend toward significance with a lower rating in the second session (p < 0.06). Peak rating for "anger" on the POMS was significantly lower after selegiline (p < 0.03).

### Metabolites and Modulators

**Figure 6:**
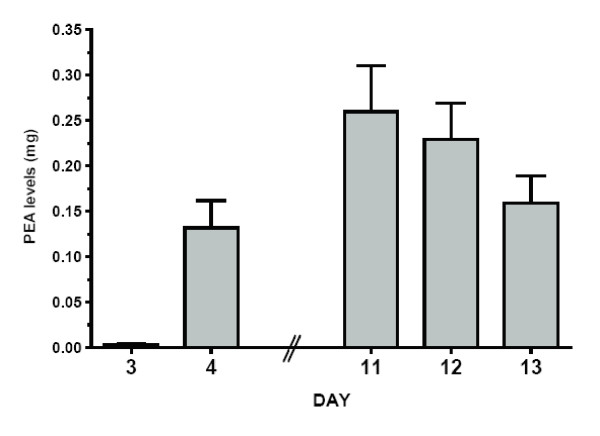
**Mean PEA Excreted in 24 Hour Urine Collection**. Selegiline started on Day 4. Error bars = SE.

Total amounts of PEA excreted in the 24-hour urine collection are shown in Figure 6 and Table 4. PEA on day 4–5 was higher than on day 3–4 (p < 0.05). After one week of selegiline administration, cocaine produced significantly greater mean PEA levels on each of the three days following the cocaine challenge (on days 11–12, 12–13 and 13–14) compared to cocaine alone (day 3–4) (p < 0.01). Mean PEA concentration on day 12–13, the second day after the second cocaine challenge, was significantly higher when compared to selegiline alone on day 4–5 (p = 0.01). No significant difference was found between PEA concentrations on days 11–12, 12–13, and 13–14.

**Table 4:** Total Amount of PEA and MHPG (mg) Excreted in the 24 Hour Urine Collection Prior to and After Application of the Selegiline Patch

**Measure**	**Day 3–4** **After Cocaine Preselegiline**	**Day 4–5** **0–24 hr Postselegiline**	**Day 11–12** **Selegiline Phase** **1^st ^Day After Cocaine**^▲^	**Day 12–13** **Selegiline Phase** **2^nd ^Day After Cocaine**^†^	**DAY 13–14** **Selegiline Phase** **3^rd ^Day After Cocaine**^‡^
PEA	0.003 ± 0.004	0.132 ± 0.095*	0.260 ± 0.171****	0.229 ± 0.145****	0.159 ± 0.104**

MHPG-S	1.44 ± 0.43	1.43 ± 0.40	1.09 ± 0.23**	1.07 ± 0.40**	1.01 ± 0.33**

Infusion started on Day 11 at 11 AM, on day 11, 2 hours after 9 AM patch application.

^▲ ^Selegiline patch applied @ 9 AM Day 12;

† Selegiline patch applied @ 9 AM Day 13;

‡ No selegiline patch was applied at 9 AM on Day 14.

* Changed from Day 3–4, p < 0.05

** Greater than Day 3–4, p < 0.01

*** Greater than Day 3–4, p < 0.001

**** Greater than Day 3–4, p < 0.0001

**Figure 7:**
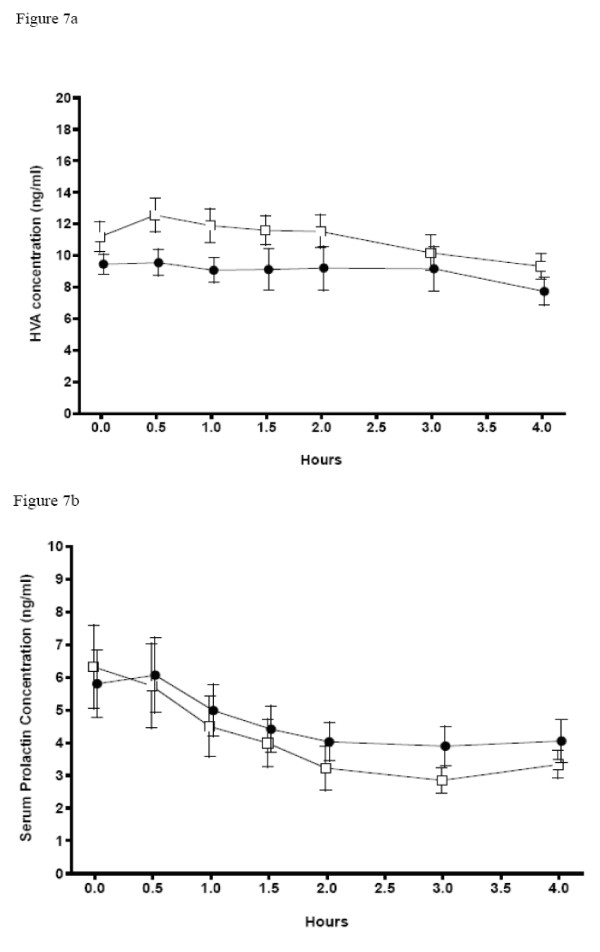
**Mean Serum Prolactin and Plasma HVA Concentrations During Cocaine Challenge Session 1 and 2**. Open Square = Session 1, Black Circle = Session 2. Error bars = SE.

Concentrations of serum prolactin (ng/ml) during the first cocaine infusion session (before selegiline) and the second session (after 5 days of selegiline; Figure 7) were not significantly different between sessions. Serum prolactin levels numerically decreased after cocaine in both challenge sessions, but because of the high variability of the data, the change within each session was not significant. Plasma HVA concentrations after cocaine challenge showed a trend (p < 0.1) toward a significant decrease at the second session following subchronic selegiline (Figure 7).

**Figure 8:**
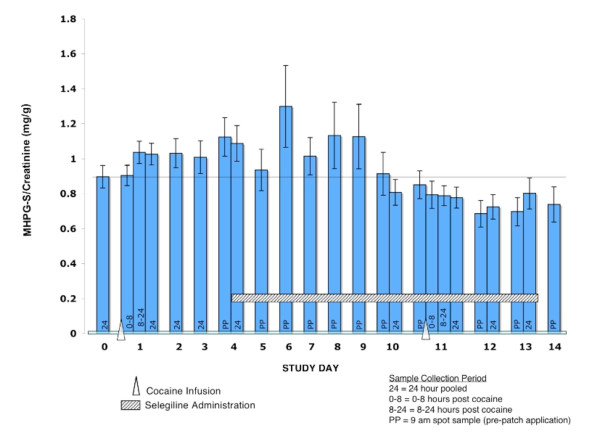
**Mean MHPG-S per Gram of Creatinine Excreted in Urine for Daily Pre-Patch Administration Urine Samples**. Error bars = SE.

Urine MHPG-S/creatinine concentration ratios increased significantly (p < 0.05) the second day after the first cocaine challenge and marginally (p < 0.1) the first and third day afterwards compared to the day before cocaine challenge. Selegiline began on day 4, and urinary MHPG S concentrations based on MHPG-S to creatinine ratios (Figure 8) did not return to baseline until day 10, the day prior to the second cocaine challenge session. However, after the second cocaine challenge the urinary MGPG-S concentrations continued to decline (though not significantly) rather than increasing. The MHPG-S concentration and MHPG-S/creatinine ratios in the three days after the second cocaine challenge were significantly lower (p < 0.01) compared to the three days after the first cocaine challenge.

### Adverse Events

One subject reported erectile dysfunction after starting selegiline and dropped out of the study. One subject experienced anxiety and one reported insomnia after beginning the patch. One subject vomited during each cocaine infusion (Session 1 and Session 2).

## Discussion

### Pharmacokinetic Measures

Plasma cocaine pharmacokinetics were unaffected by selegiline administration. After removal of the two outliers, urinary BE recovery was also unaffected. This is consistent with the similar plasma-time concentration curves found by Houtsmuller and colleagues [11] with a 40 mg cocaine challenge before and after 6 mg/24 hours of transdermal selegiline for 10 days. This absence of change in cocaine pharmacokinetics after 5 days of selegiline administration is important, because treatment trials of cocaine dependence often use urinary toxicology screens based on the presence of benzoylecgonine or quantitative benzoylecgonine concentrations as outcome measures.

Trough levels of selegiline and metabolites were similar to those reported previously after subchronic dosing [11].

### Physiologic and Subjective Effects

Physiological changes in response to cocaine were not significantly different between sessions. Houtsmuller et al. [11] have reported significant differences in heart rate and systolic blood pressure in response to a cocaine challenge after transdermal selegiline, while others [9] have not.

In our study, higher ratings of anger and tension across time were seen after cocaine alone than after cocaine and selegiline administration. Selegiline decreased craving for more cocaine between 1.5 and 4.5 hours following the cocaine infusion, a time when many users re-administer cocaine. However, cocaine craving was unchanged at the time of peak cocaine effects. The order of cocaine challenges was not randomized; that is, selegiline plus cocaine always followed cocaine alone. Because of the irreversible inhibition of selegiline requiring weeks for recovery, a balanced order design was not practical. Therefore, theoretically, this decrease in the second session could have been due to habituation or other factors. However, we and others [32-34] have not observed a change in subjective effects of cocaine with repeated within-subject dosing in previous studies. Except for a small decrease in POMS-rated anger, there were no other changes in subjective ratings and physiologic measures between cocaine challenges.

The lack of significant differences in the rewarding or stimulating effects of cocaine after transdermal selegiline in our study (e.g. "intoxication," "good drug effect,") is in contrast with reports of others [10,9,11] that found decreases in such measures as "high," "stimulated," "good effects," and "liking." These studies administered a smaller amount of cocaine (20 mg to 40 mg) in a single bolus over one minute or less. Our study administered a greater total dose of cocaine over a longer period of time, which we hoped would more closely simulate frequently reported larger doses of cocaine use. Under these conditions, selegiline did not alter the intoxicating or pleasurable effects of cocaine.

Bartzokis and colleagues [10] found that selegiline reversed the reduction in activity in the amygdala observed on PET imaging produced by a 40 mg dose of cocaine and decreased hippocampal activity both before and after cocaine challenge. Amygdala activity is postulated to be related to the rewarding effects of the drug and selegiline's reversal of cocaine's effect on activity of the amygdala may interfere with cocaine's reinforcing properties [10]. Another stimulant-like drug, methylenedioxymethamphetamine (MDMA), has been found to decrease activity in the amygdala related to some subjective effects [35]. Hippocampal activity is thought to be related to memory and craving (reviewed in [10]). Since reward and craving may have different mechanisms, it is interesting to note that the larger dose selegiline used in our study still decreased later craving but did not decrease the pleasurable effects of cocaine, as found in other studies. However, the decrease in craving may still be very important clinically and might lead to a decrease in use in motivated patients.

Orthostatic vital signs and cardiovascular monitoring, obtained during the selegiline maintenance phase, revealed no adverse cardiovascular effects from this daily selegiline dose. No significant adverse effects resulted from the administration of cocaine following selegiline administration. Selegiline used as a treatment for cocaine dependence does not appear to significantly increase risk of adverse consequences from a lapse to cocaine use during treatment, even at the higher doses commonly reported by cocaine users.

### Catecholamine Modulators and Metabolites

#### PEA

PEA has methamphetamine discriminative-stimulus and reinforcing-stimulus effects [36,37], which suggests the potential to modulate the response to stimulant drugs [38]. It is metabolized by MAO-B [17,19,18]. MAO-B inhibitors increase PEA brain concentrations [37] therefore, PEA is also an indicator of MAO-B activity. Selegiline also appears to increase the reinforcing stimulus effects of PEA [37]. Consistent with MAO-B inhibition, selegiline marginally increased urinary PEA excretion compared to cocaine alone in our study. Cocaine challenge following transdermal selegiline significantly increased urinary PEA compared to cocaine alone, as also found by Houtsmuller and colleagues [11]. Cocaine has been reported to potentiate the amphetamine-induced increase of PEA in the rat brain [39]. As selegiline is metabolized into l-methamphetamine and l-amphetamine, this synergistic effect of cocaine and selegiline on PEA increase could be expected by this mechanism also, as well as by MAO inhibition.

### Serum Prolactin and Plasma HVA

Our failure to find any differences in prolactin response has been previously reported [11]. Changes in plasma and serum concentrations of hormones and neurotransmitter metabolites are limited in their ability to reflect CNS changes. CNS changes may have been obscured by peripheral factors. Plasma HVA concentrations during cocaine challenge showed a trend towards significance for a decrease following subchronic selegiline. This would suggest that subchronic dosing of selegiline produces a more general dampening, if anything, of dopamine activity, rather than enhanced dopamine activity as predicted by the augmentation by selegiline of cocaine-induced increase in nucleus accumbens dopamine found in rodents [40]. Again, central-peripheral and regional brain differences could account for this discrepancy. Our HVA data, though not quite significant, are consistent with Newton and colleagues' [9] hypothesis that selegiline might decrease the rewarding effects of cocaine by reducing the increase in dopamine release following cocaine administration, although we did not see a rise in basal dopamine availability as they suggest. Another explanation of our marginally decreased HVA concentration is that MAO inhibition led to smaller amounts of HVA production from dopamine, regardless of dopamine changes. PEA (increased by selegiline) and MAO inhibitors themselves decrease density of some types of dopamine receptors and other monoaminergic receptors [41] and could therefore decrease dopamine-mediated effects even without change in dopamine concentrations. PEA potentiates actions of dopamine and norepinephrine, and Boulton et al. [42] suggest a co-relationship between PEA and dopamine may exist. A complex relationship between PEA, neurotransmitter release and elimination, and monoamine receptor density might explain the decrease in craving found by us and others [11] and the decrease in the rewarding effects from a smaller dose of cocaine found by others [10,9,11].

### MHPG-S

Our 4-hour cocaine infusion increased urinary MHPG-S levels for at least four days. Elevated urinary MHPG-S is thought to represent elevations in brain norepinephrine turnover, presumably due to increased release of norepinephrine into synapses with subsequent diffusion of metabolized norepinephrine out of the synapse and eventually into the systemic circulation. Therefore, a relatively brief exposure to cocaine elevated CNS norepinephrine turnover for at least three days. A direct effect of cocaine may not be the only explanation for the sustained elevations in MHPG-S excretion because the acute pharmacokinetic and dynamic effects of cocaine decline rapidly, with a half life of about 1 hour [32]. Therefore, it is more likely that cocaine (or a metabolite) induced longer lasting changes in norepinephrine regulation. Cocaine can induce long-term changes in brain neurotransmitter regulation through multiple mechanisms.

Selegiline blocks biotransformation of norepinephrine to MHPG and should decrease urinary MHPG-S levels if significant MAO-A activity is present. Cocaine alone increased MHPG-S concentrations. Following selegiline administration, MHPG-S levels gradually returned to baseline values. Selegiline blocked the cocaine-induced MHPG-S increase at the second challenge session. The significantly lower urinary MHPG-S concentrations after cocaine challenge following selegiline suggests that at this dose it may have an attenuating effect on MHPG-S production, an indication of MAO-A inhibition. This is consistent with findings in rats that transdermal selegiline also produces MAO-A inhibition in the brain even though inhibition in the gut is not clinically significant [43].

## Conclusion

Administration of a larger dose of cocaine over a longer period of time than in previously reported studies was well tolerated. This dose and duration more closely mimics a binge pattern of cocaine abuse and may yield more clinically relevant data on expected drug interactions with cocaine. With this cocaine dosing strategy, transdermal selegiline produced no significant cardiovascular adverse effects. Elevations in PEA concentrations suggest that this dose of transdermal selegiline inhibited MAO-B. The decrease in MHPG-S levels in response to cocaine challenge suggest that some MAO-A inhibition may have also taken place. The sustained increase in MHPG-S for days after the initial cocaine administration suggest mechanisms other than a direct effect of cocaine. Indicators of dopamine activity, plasma HVA and serum prolactin, suggested no increase in cocaine-induced dopamine activity following transdermal selegiline, and possibly even a small decrease in dopaminergic activity, although central-peripheral differences or variations in regional brain activity may have prevented detection of important changes. Transdermal selegiline decreased ratings of cocaine-induced anger and tension and later craving ratings but did not decrease the rewarding effects of this relatively larger dose of and longer administration of cocaine, consistent with different mechanisms for cocaine craving and reward. The largely negative effects of selegiline on response to cocaine in this laboratory study may explain the results of a study of clinical evaluation of treatment utility [4] suggesting that selegiline is not helpful as a treatment agent, despite some initial signs of clinical success. Testing response to potential therapeutic agents using cocaine doses closer to those more typically used by dependent persons is likely to better generalize to addicted populations. It is possible that a subpopulation of dependent persons, such as those with lighter use or those within a particular range of motivation to abstain, might benefit from this treatment or that yet undiscovered subtyping methods, such as genetic studies, might identify a more responsive group.

## Competing interests

The authors declare that they have no competing interests.

## Authors' contributions

DH, RTJ and JM designed the study and conducted the study. TE and PJ developed the assay methods and performed the Cocaine, MHPG, HVA, PEA assays. EL performed the selegiline assays. DH wrote the first version of the manuscript. DH and JM prepared the final manuscript. All authors read and approved the final version of the manuscript.

## Pre-publication history

The pre-publication history for this paper can be accessed here:



## References

[B1] Koob GF, Le Moal M (1997). Drug abuse: hedonic homeostatic dysregulation. Science.

[B2] Dackis CA, Gold MS (1985). New concepts in cocaine addiction: the dopamine depletion hypothesis. Neurosci Biobehav Rev.

[B3] Gerlach M, Youdim MB, Riederer P (1996). Pharmacology of selegiline. Neurology.

[B4] Elkashef A, Fudala PJ, Gorgon L, Li SH, Kahn R, Chiang N, Vocci F, Collins J, Jones K, Boardman K (2006). Double-blind, placebo-controlled trial of selegiline transdermal system (STS) for the treatment of cocaine dependence. Drug Alcohol Depend.

[B5] Barrett JS, Hochadel TJ, Morales RJ, Rohatagi S, DeWitt KE, Watson SK, DiSanto AR (1996). Pharmacokinetics and Safety of a Selegiline Transdermal System Relative to Single-Dose Oral Administration in the Elderly. Am J Ther.

[B6] Haberle D, Szoko E, Magyar K (2002). The influence of metabolism on the MAO-B inhibitory potency of selegiline. Curr Med Chem.

[B7] Barrett JS, DiSanto AR, Thomford PJ, Larsen EM, Palazzolo MJ, Morales RJ (1997). Toxicokinetic evaluation of a selegiline transdermal system in the dog. Biopharm Drug Dispos.

[B8] Haberny KA, Walsh SL, Ginn DH, Wilkins JN, Garner JE, Setoda D, Bigelow GE (1995). Absence of acute cocaine interactions with the MAO-B inhibitor selegiline. Drug Alcohol Depend.

[B9] Newton TF, Kalechstein A, Beckson M, Bartzokis G, Bridge TP, Ling W (1999). Effects of selegiline pretreatment on response to experimental cocaine administration. Psychiatry Res.

[B10] Bartzokis G, Beckson M, Newton T, Mandelkern M, Mintz J, Foster JA, Ling W, Bridge TP (1999). Selegiline effects on cocaine-induced changes in medial temporal lobe metabolism and subjective ratings of euphoria. Neuropsychopharmacology.

[B11] Houtsmuller EJ, Notes LD, Newton T, van Sluis N, Chiang N, Elkashef A, Bigelow GE (2004). Transdermal selegiline and intravenous cocaine: safety and interactions. Psychopharmacology (Berl).

[B12] Amin F, Davidson M, Davis KL (1992). Homovanillic acid measurement in clinical research: a review of methodology. Schizophr Bull.

[B13] Peyrin L (1990). Urinary MHPG sulfate as a marker of central norepinephrine metabolism: a commentary. J Neural Transm Gen Sect.

[B14] Smookler S, Bermudez AJ (1982). Hypertensive crisis resulting from an MAO inhibitor and an over-the-counter appetite suppressant. Ann Emerg Med.

[B15] Darmani NA (1996). Differential potentiation of L-tryptophan-induced head-twitch response in mice by cocaine and sertraline. Life Sci.

[B16] Brewer C (1993). Treatment of cocaine abuse with monoamine oxidase inhibitors. Br J Psychiatry.

[B17] Johnston JP (1968). Some observations upon a new inhibitor of monoamine oxidase in brain tissue. Biochem Pharmacol.

[B18] Henry DP, Russell WI, Clemens JA, Plebus LA, Boulton AA, Juorio AV, Downer RGH (1988). Phenylethylamine and p-tyramine in the extracellular space of the rat brain: quantification using a new radioenzymatic assay and in situ microdialysis. Trace Amines: Comparative and Clinical Neurobiology.

[B19] Paterson IA, Juorio AV, Boulton AA (1990). 2-Phenylethylamine: a modulator of catecholamine transmission in the mammalian central nervous system?. J Neurochem.

[B20] Scheinin M, Karhuvaara S, Ojala-Karlsson P, Kallio A, Koulu M (1991). Plasma 3,4-dihydroxyphenylglycol (DHPG) and 3-methoxy-4-hydroxyphenylglycol (MHPG) are insensitive indicators of alpha 2-adrenoceptor mediated regulation of norepinephrine release in healthy human volunteers. Life Sci.

[B21] Everhart ET, Jacob P, Mendelson J, Jones RT (1999). The synthesis of deuterium-labeled cocaine, cocaethylene and metabolites. J Labelled Cpd Radiopharm.

[B22] Rohatagi S, Barrett JS, DeWitt KE, Morales RJ (1996). Multiple dose pharmacokinetics and dose proportionality of selegiline and metabolites in healthy males following transdermal administration (Abstr). Pharm Res.

[B23] Simon SL, Richardson K, Dacey J, Glynn S, Domier CP, Rawson RA, Ling W (2002). A comparison of patterns of methamphetamine and cocaine use. J Addict Dis.

[B24] Alberta Health Service Beyond the ABCs – Cocaine. http://www.aadac.com/87_417.asp.

[B25] Arnett CD, Fowler JS, MacGregor RR, Schlyer DJ, Wolf AP, Langstrom B, Halldin C (1987). Turnover of brain monoamine oxidase measured in vivo by positron emission tomography using L-[11C]deprenyl. J Neurochem.

[B26] McNair DM, Lorr M, Droppleman LF (1971). EITS Manual for the Profile of Mood States.

[B27] Jacob P, Elias-Baker BA, Jones RT, Benowitz NL (1987). Determination of benzoylecgonine and cocaine in biologic fluids by automated gas chromatography. J Chromatogr.

[B28] Minegishi A, Ishizaki T (1984). Rapid and simple method for the simultaneous determination of 3,4-dihydroxyphenylacetic acid, 5-hydroxyindole-3-acetic acid and 4-hydroxy-3-methoxyphenylacetic acid in human plasma by high-performance liquid chromatography with electrochemical detection. J Chromatogr.

[B29] Jacob P, Wilson M, Yu L, Mendelson J, Jones RT (2002). Determination of 4-hydroxy-3-methoxyphenylethylene glycol 4-sulfate in human urine using liquid chromatography-tandem mass spectrometry. Anal Chem.

[B30] Gibaldi M, Perrier D, Eds (1982). Pharmacokinetics.

[B31] (1999). WinNonlin Pro.

[B32] Harris DS, Everhart ET, Mendelson J, Jones RT (2003). The pharmacology of cocaethylene in humans following cocaine and ethanol administration. Drug Alcohol Depend.

[B33] Walsh SL, Sullivan JT, Preston KL, Garner JE, Bigelow GE (1996). Effects of naltrexone on response to intravenous cocaine, hydromorphone and their combination in humans. J Pharmacol Exp Ther.

[B34] Evans SM, Walsh SL, Levin FR, Foltin RW, Fischman MW, Bigelow GE (2001). Effect of flupenthixol on subjective and cardiovascular responses to intravenous cocaine in humans. Drug Alcohol Depend.

[B35] Gamma A, Buck A, Berthold T, Liechti ME, Vollenweider FX (2000). 3,4-Methylenedioxymethamphetamine (MDMA) modulates cortical and limbic brain activity as measured by [H(2)(15)O]-PET in healthy humans. Neuropsychopharmacology.

[B36] Greenshaw AJ (1984). beta-Phenylethylamine and reinforcement. Prog Neuropsychopharmacol Biol Psychiatry.

[B37] Bergman J, Yasar S, Winger G (2001). Psychomotor stimulant effects of beta-phenylethylamine in monkeys treated with MAO-B inhibitors. Psychopharmacology (Berl).

[B38] Colpaert FC, Niemegeers CJ, Janssen PA (1980). Evidence that a preferred substrate for type B monoamine oxidase mediates stimulus properties of MAO inhibitors: a possible role for beta-phenylethylamine in the cocaine cue. Pharmacol Biochem Behav.

[B39] Karoum F, Wolf ME, Mosnaim AD (1997). Effects of the administration of amphetamine, either alone or in combination with reserpine or cocaine, on regional brain beta-phenylethylamine and dopamine release. Am J Ther.

[B40] Schiffer WK, Azmoodeh M, Gerasimov M, Volkow ND, Fowler JS, Dewey SL (2003). Selegiline potentiates cocaine-induced increases in rodent nucleus accumbens dopamine. Synapse.

[B41] Paetsch PR, Greenshaw AJ (1993). 2-Phenylethylamine-induced changes in catecholamine receptor density: implications for antidepressant drug action. Neurochem Res.

[B42] Boulton AA, Juorio AV, Paterson IA (1990). Phenylethylamine in the CNS: effects of monoamine oxidase inhibiting drugs, deuterium substitution and lesions and its role in the neuromodulation of catecholaminergic neurotransmission. J Neural Transm Suppl.

[B43] Wecker L, James S, Copeland N, Pacheco MA (2003). Transdermal selegiline: targeted effects on monoamine oxidases in the brain. Biol Psychiatry.

